# Distribution of the Emetic Toxin Cereulide in Cow Milk

**DOI:** 10.3390/toxins13080528

**Published:** 2021-07-28

**Authors:** Veronika Walser, Markus Kranzler, Corinna Dawid, Monika Ehling-Schulz, Timo D. Stark, Thomas F. Hofmann

**Affiliations:** 1 Food Chemistry and Molecular Sensory Science, Department of Molecular Life Sciences, School of Life Sciences, Technical University of Munich, Lise-Meitner-Str. 34, 85354 Freising, Germany; veronika.walser@tum.de (V.W.); corinna.dawid@tum.de (C.D.); thomas.hofmann@tum.de (T.F.H.); 2 Institute of Microbiology, Department of Pathobiology, University of Veterinary Medicine Vienna, Veterinärplatz 1, 1210 Vienna, Austria; Markus.Kranzler@vetmeduni.ac.at (M.K.); monika.ehling-schulz@vetmeduni.ac.at (M.E.-S.)

**Keywords:** *B. cereus*, lipids, interaction, food safety, LC-MS/MS

## Abstract

*Bacillus cereus* is frequently associated with food-borne intoxications, and its emetic toxin cereulide causes emesis and nausea after consumption of contaminated foods. The major source for contamination is found within contaminated raw materials containing the highly chemically resistant cereulide, independent of vegetative bacteria cells. Up to date, non-existing removal strategies for cereulide evoke the question of how the toxin is distributed within a food sample, especially cow milk. Milk samples with different milk fat contents were incubated with purified cereulide, separated by centrifugation into a lipid and an aqueous phase, and cereulide was quantified in both fractions by SIDA-LC-MS/MS. By artificially increasing the milk fat content from 0.5% to 50%, the amount of cereulide recovered in the lipid phase and could be augmented from 13.3 to 78.6%. Further, the ratio of cereulide increased in the lipid phase of milk with additional plant-based lipid (sunflower oil) to 47.8%. This demonstrated a clear affinity of cereulide towards the hydrophobic, lipid phase, aligning with cereulide’s naturally strong hydrophobic properties. Therefore, an intensified cereulide analysis of lipid enriched dairy products to prevent severe cereulide intoxications or cross-contamination in processed foods is suggested.

## 1. Introduction

*Bacillus cereus* is an ubiquitous, endospore forming, facultative anaerobe pathogen, commonly associated with food-borne intoxications, occurring typically in the form of gastrointestinal diseases and emesis [1]. The bacterium itself is often found in raw materials, but can also be found in processed foods, such as wheat-based, dairy and meat products, and processed vegetables or spices, and might possess the ability to produce enterotoxins or the emetic toxin, rendering it a severe food-borne pathogen [2,3,4,5]. Emesis is induced 0.5–6 h after consumption of contaminated products by the preformed emetic toxin cereulide, a cyclic dodecadepsipeptide consisting of twelve alternating *α*-amino acids and *α*-hydroxy acids [6]. With its complex chemical structure (l-*O*-Val-l-Val-d-*O*-Leu-d-Ala)_3_ [6,7,8,9,10], cereulide is characterized to be chemically resistant against wide ranges of temperature and pH values as well as enzymes, and subsequently poses an enormous safety hazard for the food industry [10,11,12,13]. After consumption of contaminated foods, some patients showed symptoms of strong pain and vomiting, or even exposed a more severe course of disease with liver failure, encephalopathy, or metabolic acidosis [14,15,16,17,18,19,20]. By recently using cereulide in a porcine model, cereulides ability to cross the blood brain barrier could be established, and therefore, a subsequent disturbance of the potassium content in the cerebrospinal fluid was suggested. This might partially explain the cerebral effects reported in human intoxication cases [21].

In a recent study on the *B. cereus* growth and cereulide production in various food-based matrices, cereal-based food products were classified highest in their ability to support cereulide production, followed by dairy products, which showed intermediate cereulide production [22]. Various research groups performed surveys on the *B. cereus* prevalence in dairy products in their home countries and found high contamination rates of apathogenic as well as pathogenic *B. cereus*, not only in raw milk but also in pasteurized and ultrahigh-temperature treated milk products from their respective investigation area, thus highlighting the considerable incidence of *B. cereus* as possible food-pathogen [23,24,25,26,27]. Contamination of milk and pasteurized dairy products was reported to most likely occur by entry of heat stable *B. cereus* spores into the raw milk material, but bacterial growth and distribution should also be closely monitored along the processing line during handling, post-pasteurization processing, or storage steps [28,29,30,31].

While vegetative *B. cereus* cells can be eliminated during food processing, up to now, there is no removal strategy for the chemically resistant toxin cereulide. Therefore, this study focused on the localization of cereulide within a milk matrix to uncover possible distribution or interaction processes, with the overall objective to highlight critical parameters, and help the food industry to optimize their sampling procedure for cereulide detection.

## 2. Results and Discussion

### 2.1. Distribution of Cereulide within a Milk Matrix and Milk Matrix with Increased Lipid Contents

Missing removal strategies for cereulide elicit the urgent question of what happens to the toxin once it enters the food processing line, either by contamination with emetic *B. cereus* in the raw material and subsequently producing cereulide, or possibly from additives that may not contain vegetative bacteria cells anymore, but might still contain the resistant and persistent toxin cereulide.

To clarify this question, commercially available pasteurized milk with varying milk fat contents was spiked with the target analyte cereulide and incubated at room temperature. After centrifugal separation of the cereulide-spiked milk samples into the lipid phase and the remaining aqueous phase, LC-MS/MS quantitation of cereulide was performed. To ensure that the toxin would not show unspecific interaction with employed plastic surfaces, the safe-lock tubes—used during the incubation of the milk samples—were separately incubated with cereulide in aqueous DMSO (10%, *v/v*), and mass spectrometrically analyzed for cereulide possibly attached to their plastic surface. This led to the conclusion that the amount of cereulide detained by the safe-lock tubes could be neglected in all further evaluations (data not shown).

**Figure 1:**
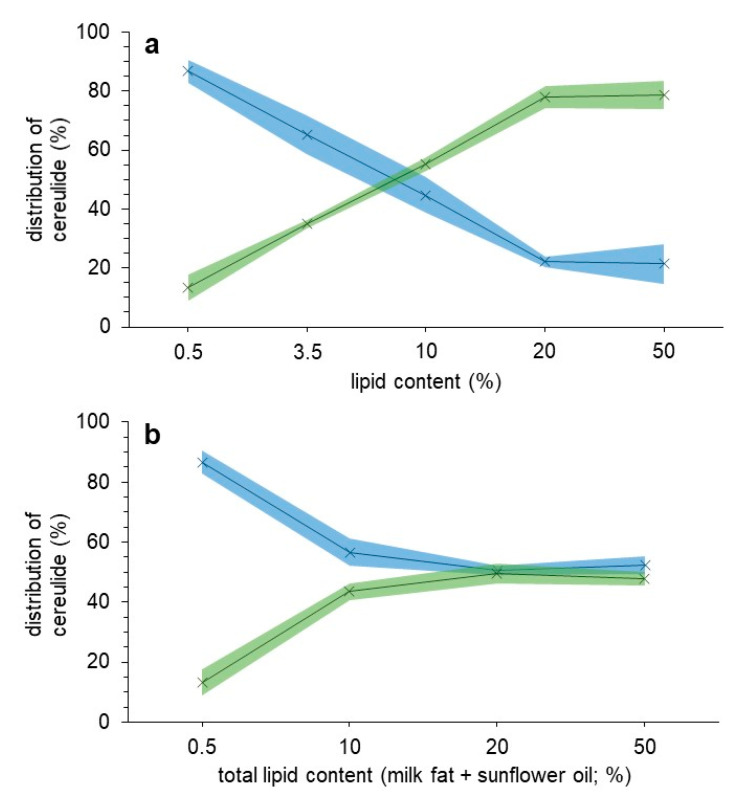
Percentile distribution of cereulide between the aqueous (blue) and the lipid phase (green) in milk matrices with (**a**) variable milk fat content and (**b**) 0.5% milk fat with additional sunflower oil. Measurement values as well as deviation were linearly connected for displaying tendency.

For commercially obtained milk with a milk fat content of 0.5%, 86.7 ± 3.9% of the spiked cereulide was located in the aqueous phase, while only 13.3 ± 4.3% could be found in the lipid phase (Figure 1a). When investigating milk with 3.5% fat content, an increase to 34.9 ± 1.5% cereulide in the lipid phase could be observed, giving a first hint towards the augmented affinity of cereulide towards milk fat. With stepwise increasing milk fat content in the analyzed samples to 10, 20, or 50%, the amount of cereulide detected in the lipid phase increased to a total of 78.6 ± 4.9% (50% milk fat; Figure 1a). This shift in favor of the lipid phase also fit well together with the hydrophobic properties of cereulide that obviously led to an emigration from the aqueous fraction.

Instead of stepwise increasing the milk fat content, in a second experiment, milk with 0.5% fat was laced with commercially available sunflower oil to reach an overall lipid content of 10, 20, or 50%, and the distribution of cereulide monitored analogously. At a lipid content of 50% in the sample, 47.8 ± 2.2% of cereulide could be analyzed in the lipid phase, while 52.2 ± 3.0% of cereulide was found present in the aqueous fraction (Figure 1b).

### 2.2. Discussion and Conclusions

Overall, in both experiments, a tendency of cereulide enrichment in the lipid phase of the respective sample could be observed, which is in alignment with the high hydrophobicity of the toxin. Interestingly, a variance in the influence of additional milk fat or sunflower oil could be monitored. With increasing the milk fat content from 0.5 to 50%, the percentage of cereulide located in the lipid phase augmented from 13.3 ± 4.3 to 78.6 ± 4.9%, while at the same time, additional sunflower oil only effectuated an increase up to 47.8 ± 2.2%. The source for this discrepancy might originate in the better homogenization of the milk sample with additional milk fat rather than sunflower oil, which showed a very quick phase separation, or might be induced by the different chemical composition of the lipid sources. However, the exact reasons for this strong deviation were not further investigated here. The main goal of this study was to highlight the high safety hazard of a possible enrichment of cereulide into food with a high lipid content. Considering the findings in this study, a cross contamination of cereulide to dairy products could arise, even though *B. cereus* growth coupled with cereulide production is described to mainly occur in foods with a high carbohydrate content (e.g., rice or pasta dishes), as were recently reported for contaminated pasta dishes with cereulide levels of up to 6198.17 (±162.09) ng/g [22,32].

In conclusion, these experiments show a heightened affinity of the food toxin cereulide towards the lipid components rather than the aqueous fraction in the milk samples. This knowledge might also be applicable to other dairy products, especially those with enriched milk fat content, and highlights the hazard of cereulide enrichment along the food production line. An accumulation of cereulide in intermediate lipid-enriched products, without the presence of vegetative bacteria cells, for example, can lead towards contamination of the final food product, due to negative *B. cereus* testing and a possibly missing cereulide analysis during the quality control process. Therefore, this study suggests an emphasized control of especially lipid enriched dairy products or processed foods containing dairy products for the emetic toxin cereulide itself, even if no vegetative *B. cereus* cells could be detected or bacteria growth is unlikely.

To fully implement these preliminary findings in future cereulide analysis in the dairy industry, a broad variety of naturally occurring *B. cereus* or cereulide contaminated samples, respectively, needs to be investigated. This step will be crucial for testing the behavior of the naturally present cereulide within the milk emulsion, building a strong evidence-based database on cereulide quantitation and distribution, and exploring the sensitivity limitations entailed by this approach.

## 3. Materials and Methods

### 3.1. Chemicals and Samples

The following compounds and samples were obtained commercially: dimethyl-sulfoxide (≥99%; DMSO), DMSO-*d*_6_, and MeOH-*d*_4_ from Sigma-Aldrich (Steinheim, Germany); ethanol (HPLC grade) from Honeywell (Seelze, Germany); ^13^C_6_-cereulide (>95%) from Chiralix (Nijmegen, Netherlands); and sunflower oil, cow milk (pasteurized, 0.5% fat content), and cow milk (pasteurized, 3.5% fat content) from a local supermarket.

Cereulide was obtained from a *B. cereus* culture of the emetic reference strain F4810/72. Cultivation, extraction, and isolation were performed as reported recently [33]. For all experiments, solutions of cereulide (200 µg/mL; DMSO) and ^13^C_6_-cereulide (1 µg/mL; EtOH) were applied. Concentration of cereulide and ^13^C_6_-cereulide was determined via q-^1^H-NMR spectroscopy and diluted accordingly.

### 3.2. Sample Workup

Aliquots of the store-bought cow milk samples (900 µL, each) were put in 2 mL safe-lock plastic centrifugal tubes (Eppendorf, Hamburg, Germany), spiked with cereulide (100 µL; 200 µg/mL; DMSO), and incubated for three hours whilst shaking (800 rpm, RT). The DMSO content of 10% within the samples prevents precipitation of the non-polar cereulide in the aqueous environment. After incubation, the samples were centrifuged to achieve separation of the aqueous and lipid phase (30 min, 13,200 rpm, 4 °C). The solidified lipid phase was separated with a disposable plastic spatula and transferred to a second safe-lock tube. All samples were freeze-dried, and the cereulide separately re-extracted from both sample parts (aqueous and lipid phase). Extraction was performed with EtOH (1 mL, 10 min, 800 rpm, RT), followed by a dilution with ethanol (1/10; *v/v*), including the addition of ^13^C_6_-cereulide as an internal standard (100 µL; 1 µg/mL; EtOH). The samples were then utilized for UPLC-MS/MS analysis.

For samples with enriched milk-fat content (10%, 20%, 50%), the accordingly needed amount of milk fat was isolated from milk (3.5% fat content) by means of centrifugation, as described above. The respectively solidified amount of milk-fat was removed with a disposable plastic spatula and added to a second aliquot of milk to be analyzed (3.5% fat content). The milk samples with enriched milk-fat content were then homogenized by alternating vortexing and shaking (30 min; 800 rpm; RT). The further sample work up was performed as described above.

Samples with additional plant-based oil content were spiked with the particularly calculated amount of sunflower oil to reach an overall lipid content of 10, 20, or 50%, respectively. As the primary sample material, milk with 0.5% milk-fat content was used. Sample homogenization was attempted by alternating vortexing and shaking (30 min; 800 rpm; RT), but ultimately could not be fully accomplished due to quick phase formation of the sunflower oil. The further sample work up was as described above, with the slight alteration, that the separated liquid lipid phase was removed with a disposable pipette.

All samples were prepared as biological triplicates and analyzed via UPLC-MS/MS as technical triplicates.

### 3.3. Technical Data on Mass Spectrometry and NMR-Spectroscopy

The quantitation of cereulide was performed with a sample aliquot of 2 µL, each, on a Waters Xevo TQ-S mass spectrometer (Waters, Manchester, UK) coupled to an Acquity UPLC i-class core system (Waters, Manchester, UK) comprising a binary solvent manager, sample manager, and column oven [32]. The system was run with the MassLynx 4.1 SCN 813 Software (Waters, Manchester, UK), and data processing and analysis were executed with TargetLynx (Waters, Manchester, UK). For quantitation, the ammonium adducts of cereulide and ^13^C_6_-cereulide were utilized in the multiple reaction monitoring (MRM) mode with their transitions being analyzed for a period of 25 ms, and were acquired according to literature [32]: cereulide (*m/z* 1170.7 → qualifier, *m/z* 172.2, 314.2; quantifier, *m/z* 357.2) and ^13^C_6_-cereulide (*m/z* 1176.7 → qualifier, *m/z* 173.2, 316.2; quantifier, *m/z* 358.2). Quantitation was performed with plotting the peak area ratios of analyte to the internal standard against the concentration ratios of analyte to the internal standard of mixtures of cereulide (0.1–1000 ng/mL in EtOH) and ^13^C_6_-cereulide (100 ng/mL) as the internal standard, which were analyzed in triplicates by means of UPLC-MS/MS. By applying linear regression (origin excluded), the calibration curve was obtained as y=2.1827x+0.1397 with $R^{2}=0.9990$. Recovery rates of cereulide for all analyzed sample sets were between 99.5 and 119.9% of spiked cereulide. Due to identical technical set-up, the sensitivity of this UPLC-MS/MS quantitation method was expected to be similar to the recently reported cereulide levels of 0.45 ng/g and 50 ng/g [21,32] and was not further investigated.

Analysis was performed in the positive electrospray ionization (ESI) mode and quantitative calibration mode applying the ion source parameters as follows: capillary voltage, +2.0 kV; sampling cone, 30 V; source offset, 20 V; source temperature, 150 °C; desolvation temperature, 650 °C; cone gas, 150 L/h; desolvation gas, 1100 L/h; collision gas flow, 0.15 mL/min; and nebulizer gas flow, 7.0 bar. The mass spectrometer was calibrated in the range from *m/z* 40 to 1963, using a solution of phosphoric acid (0.1% in MeCN). Chromatography was performed on a 2.1 × 50 mm, 1.7 µm, UPLC BEH C18 column (Waters, Manchester, UK), with a flowrate of 1 mL/min of aqueous HCOONH_4_ (10 mmol, Solvent A) and MeCN with 0.3% HCOOH (Solvent B) at 55 °C, staring at 95% B, increasing it to 99% B in 0.5 min, holding isocratically for 0.3 min, decreasing to 95% within 0.1 min, and holding at 95% for 0.1 min.

The quantitative proton Nuclear Magnetic Resonance (q-^1^H-NMR) spectrum of cereulide and ^13^C_6_-cereulide were recorded using a 400 MHz Avance III spectrometer with a Broadband Observe BBFO plus (Bruker, Rheinstetten, Germany) according to reference [34]. The chemical shift was referenced to the solvent signal of DMSO-*d*_6_ and MeOH-*d*_4_, respectively. Data acquisition, processing, and evaluation were performed with the Topspin Software Version 4.0.7 (Bruker, Rheinstetten, Germany).

## Data Availability

Data is contained within the article.
